# GPs' views on transfer of information about terminally ill patients to the out-of-hours co-operative

**DOI:** 10.1186/1472-684X-8-19

**Published:** 2009-12-22

**Authors:** Bart Schweitzer, Nettie Blankenstein, Maartje Willekens, Elmi Terpstra, Paul Giesen, Luc Deliens

**Affiliations:** 1 Department of General Practice, and EMGO Institute of Health and Care Research, VU University Medical Center, Amsterdam, The Netherlands; 2 Centre for Quality of Care Research, Radboud University, Nijmegen, The Netherlands; 3 Department of Public and Occupational Health, and EMGO Institute of Health and Care Research, VU University Medical Centre, Amsterdam, The Netherlands; 4 End-of-Life Care Research Group, Vrije Universiteit Brussels, Belgium

## Abstract

**Background:**

In the Netherlands, the increase in of out-of-hours care that is provided by GP co-operatives is challenging the continuity of care for the terminally ill in general practice. Aim of this study is to investigate the views of general practitioners (GPs) on the transfer of information about terminally ill patients to the GP co-operatives. GPs were asked to give their view from two different perspectives: as a GP in their daily practice and as a locum in the GP co-operative.

**Methods:**

Retrospective web based questionnaire sent to all 424 GPs in the Amsterdam region.

**Results:**

With a response rate of 42%, 177 physicians completed the questionnaire. Transfer of information to the GP co-operative about most of their terminally ill patients was reported by 82% of the GPs and 5% did not do so for any of their patients. A faster than foreseen deterioration of the patient's situation was the most frequently reported reason for not transferring information.

Of those who transferred information to the GP co-operative, more than 95% reported that they provided information about the diagnosis and terminally ill status of the patient. Information about medication, patient wishes regarding treatment, and prognosis was reported by respectively 90%, 87%, and 74% of the GPs. Less than 50% of the GPs reported that they transferred information about the patient's awareness of both the diagnosis and the prognosis, about the psychosocial context, and intolerances.

In their role as locum, over 90% of the GPs wanted to receive information about the diagnosis, the terminally ill status of the patient, the medication and the patient's wishes regarding treatment.

**Conclusions:**

Although most GPs reported that they transferred information about their terminally ill patients to the GP co-operative, the content of this information varies considerably. Only 21% of the GPs, working out of hours as a locum, were satisfied with the quality of the information transferred.

## Background

In the Netherlands, 60% of all patients dying with cancer or a terminal chronic disease, dies at home[1,2]. On average, per year, a general practitioner (GP) is responsible for the care of five to six patients with cancer in a terminal phase[3]. Therefore, primary palliative care is regarded as an important task of the GP in the Netherlands. GPs do not only take care of their patients during office hours, but until 1999 most patients also received out-of-hours palliative care from their own GP, including care during the weekends [1]. Until the 1960s, many Dutch GPs personally took care of their own patients out-of-hours. As a consequence, GPs were on call most of the time. Subsequently, more and more GPs formed small rota groups of five to ten GPs, in which they were on call for each other's patients. However, from 1999 on, GPs in the Netherlands have reorganised their out-of-hours care from rota groups to larger scale GP co-operatives with 40-400 GPs taking care of populations ranging from 50.000-700.000 inhabitants [4,5] These co-operatives now provide out-of-hours GP care for more than 90% of the Dutch population. They are organised by and responsible to a board of local GPs.

In general, GPs and patients seem to be positive about these GP co-operatives, [6] but some patients have expressed concerns regarding the care that complex, time consuming, palliative care patients receive[6,7]. In palliative care continuity of the care is considered to be quite important by most patients; when personal continuity is not possible, information must be transferred in order to ensure optimal out-of-hours care. The restructuring of GP care during the out-of-hours period is challenging the continuity of care that is needed in end-of- life care situations. The new out-of-hours arrangements have made informational continuity of crucial importance [8].

However, one of the major problems in palliative care appears to be the poor communication about terminally ill patients between the GPs and the co-operatives. Several studies in the United Kingdom (UK) have reported that few GPs report that they routinely hand over information about their palliative care patients to their GP co-operatives. This results in care that is often not comprehensive, problems in symptom control and unnecessary hospital admissions [9]. Moreover, it can leave patients and their carers confused, and inadequately supported [9-14]. Although, in general, GPs in the UK are satisfied with current out-of-hours arrangements, there is less satisfaction in the inner-city areas [15]. We did not find any studies focusing on the views of GPs on the transfer of information, specifically about terminally ill patients.

In the process of designing a new out-of-hours protocol for palliative care we wanted to analyse the experiences of GPs with the transfer of information from two perspectives: from their position as a GP caring for their terminally ill patients, and from their perspective as a locum for the GP co-operative

This paper reports on the views of GPs working in a big city on the transfer of information for terminally ill patients from GP practices to GP co-operatives, and vice versa. The research questions were:

1. How many GPs report that they transfer information about their terminally ill patients to the GP co-operative? And, if they do not, what are their reasons?

2. What information do GPs transfer?

3. In their role as locum, what information do GPs want to receive?

4. How satisfied are GPs with the feedback report on their patients from the locum?

The Ethics Board of the Radboud University, Nijmegen was informed about the study, but the study did not undergo formal ethics review.

## Methods

A retrospective survey was carried out among all GPs (N = 424) in the region of Amsterdam in October 2006, using a web based questionnaire. Names and contact details were obtained from the Amsterdam GP co-operative. This co-operative has been in place since 2000. All GPs participate in 8 out-of-hours GP posts belonging to the Amsterdam GP co-operative. Most of them actually work their shifts as a locum for this co-operative.

In this study, a terminally ill patient is defined as a patient who is in the last phase of life, for whom no further cure is possible and life expectancy is limited, independent of the underlying illness [16].

### Measurement instruments

We used a questionnaire concerning the quality of terminal care provided by the GP co-operative. The questionnaire was based on a review of the literature.

A panel of experts in palliative care assessed a concept questionnaire and amendments were made. The result was commented on by a second panel of experienced GPs and agreed upon in a meeting with GPs and specialists. It was then piloted with 239 GPs and after minor changes a final version was prepared.

The questionnaire contained open and multiple-choice questions. For the study described in this paper, we used the questions about the transfer of information transfer from GP to GP co-operative and vice versa, and the questions assessing the importance of the information that is transferred.(See Appendix [Additional file 1]: the survey was conducted in Dutch, the Appendix is an English translation)

Data on GP characteristics were obtained from the annual report of the Amsterdam GP co-operative.

### Procedure

Of all the 424 eligible GPs, 387 received an e-mail inviting them to fill in a questionnaire on a website, and the remaining 37 GPs, who had no e-mail address, received a postal questionnaire. Those who received the e-mail request also received a specific code, which they could only fill in once. In this questionnaire it was not possible to leave questions unanswered. Two reminders were sent, in an attempt to achieve a higher response rate, including a multiple-choice question about the reasons for non-response.

### Data analyses

Data were analysed with Microsoft Excel and SPSS 12.0. Answers on a 5-point scale were converted to a 3-point scale. For example answers 1 and 2 (very unimportant and unimportant) were clustered under the denominator "unimportant", answers 4 and 5 (important and very important) were clustered under the denominator "important" and answer 3 remained unchanged under the denominator "neutral".

Chi-square tests were used to analyse differences in GP characteristics between responding GPs versus [1] all GPs in Amsterdam, [2] GPs who stated that they were personally available to provide care for their terminally ill patients during the out-of-hours period (GPs personally available), and [3] GPs who stated that they did not often transfer information.

## Results

The response rate for the questionnaire was 42%. The e-mail questionnaire was completed by 175 GPs and the postal version by four GPs, two of whom were excluded from the data analysis due to incomplete answers. Hence, the results are based on the responses of 177 GPs. Of the 249 GPs who did not fill in the questionnaire, 33 (13.3%) answered by e-mail and gave their reasons for non-participation (more than one answer allowed): 25 reported that they were too busy, or forgot to reply, eight stated that they did not like web based questionnaires, and eight indicated that the questionnaire was too long.

None of the GPs reported lack of interest in the subject.

Of the respondents 61% were male, with a mean age of 49.6 years (SD 8.1). 42.3% worked either in a group practice or a community health centre, 32.6% worked in a duo-practice and 25.1% worked in a single-handed practice. The majority (63.4%) was working for three or four days a week and 34.9% were working full-time.

The GPs who responded did not differ significantly from the total population of GPs in the region. (Table 1)

**Table 1:** GP characteristics

	All GPs in Amsterdam (N = 424)	All Responding GPs (N = 177)	P values^1^	Responding GPs personally available (N = 123)	P Values^2^	Responding GPs not frequently transferring information (N = 31)	P Values^3^
Mean Age		49.6 (range 33-66)		50.8		52.9	
Sex							
Male	246 58%	108 61%	p = 0.586	78 65%	p = 0.104	28 90% ()	p = 0.002
Female	198 42%	69 39%		45 35%		3 10%	

Practice							
Single-handed practice	98 23%	44 25%	p = 0.71	32 26%	p = 0.908	11 38.7% ()	p = 0.001
Duo practice	144 34%	58 33%		38 32.5%		13 41.9%	
Group practice	182 43%	75 42%		53 41.5%		7 19.4%	

Working hours							
3-4 days/week	246 58%	112 63%	p = 0,805	77 626	p = 0307	14 45.2%	p = 0.002
4 days or more/week	144 34%	62 35%		45 36.6%		17 54.8%	

1 = difference between all responding GPs and all GPs

2 = difference between GPs personally available and all responding GPs

3 = difference between GPs not frequently transferring information and all responding GPs

Of the respondents, 70% stated that they were personally available to provide care for their terminally ill patients during out-of-hours periods, even if they also made use of the GP co-operative. (GPs personally available). In this sub-group male GPs are more often available than female GPs but this difference is not statistically significant (p = 0.104), neither are the differences in practice form nor working hours.

### Transfer of information to the GP cooperative

Of the respondents, 82.3% reported that they transferred information to the GP co-operative about most of their terminal ill patients, 12.6% did this in approximately half of the cases, and 5.1% rarely or never did so. The group of GPs not often transferring information was more often male (90%, p = 0,001), working in a single-handled practice (38.7%, p = 0,002) and working four or more days a week (54.8% (p = 0,001). The GPs personally available reported in 78.9% that they transferred information usually, 14.6% did this in half of the cases and 6.6% rarely or never. (p = 0,208)

Table 2 shows the reasons for not transferring information. The most frequently reported reason was a faster than foreseen deterioration of the patient's medical condition (48.6%). In the category "other reasons", four GPs answered that they "did not expect problems with this patient"; two GPs did not transfer information because they were too busy, and one did not do so because he always left the information at the patient's home.

**Table 2:** Reported reasons for not transferring information* (N = 177)

	Responding GPs (N = 177)	Responding GPs personally available (N = 123)	P values ^1^	Responding GPs not frequently transferring information (N = 31)	P values ^2^
1. Deterioration of patient's medical condition faster than foreseen	86 48.6%	62 50.4%	p = 0.757	15 48.8%	p = 0.084
2. Forgotten	66 37.1%	49 39.8%	p = 0.957	14 45,2%	p = 0.557
3. I am always personally available	38 21.7%	33 26.8%	p = 0.283	17 54,8%	p = 0.015
4. Patient currently dismissed from hospital	35 20.0%	27 22.0%	p = 0.647	3 9,7%	p = 0.180
5. Too much administration	12 6.9%	10 8.1%	p = 0.659	6 19,4%	p = 0.022
6. Other reasons	8 4.6%	8 6.5%	p = 0.452	1 3,2%	p = 0.744

* (more than one answer possible)

1 = difference between GPs personally available and all responding GPs

2 = difference between GPs not frequently transferring information and all responding GPs

In the group of GPs not often transferring information the most reported reason for not doing so was that they were personally available (54,8%).

The GPs personally available did not differ significantly from the other respondents.

### Content of the transferred information

More than 90% of the GPs reported the diagnosis, the terminally ill status of the patient and patient's medication. (Table 3) Information about the treatment wished by the patient and the prognosis was transferred by respectively 87% and 74%. Information about whether or not the patient knows about the diagnosis and prognosis, the psychosocial context, intolerances for medication, and the content of the previous five contacts was transferred by less than 50% of the GPs.

**Table 3:** Reported content of information and assessment of information by the locum (N = 177)

Information	Information transferred by GP (%)	Assessment of information by locum		
		Unimportant(%)	Neutral(%)	Important(%)
1. Diagnosis	96.6	-	1.1	98.9
2. Terminally ill patient	95.4	-	4.0	96.0
3. Medication	90.9	1.7	2.9	95.4
4. Desired patient treatment (eg. pain treatment)	87.4	0.6	6.9	92.6
5. Prognosis	74.3	5.1	18.3	76.6
6. Relevant changes in disease process	68.0	1.7	10.3	88.0
7. Patients wishes regarding end-of- life care	67.4	3.4	10.9	85.7
8. List of problems	61.1	10.9	29.7	59.4
9. Private telephone number GP	52.0	28.0	35.4	36.6
10. Patient's awareness of prognosis	41.4	8.0	22.3	69.7
11. Psychosocial context	38.9	4.6	30.3	65.1
12. Intolerances for medication	37.1	14.3	30.9	54.9
13. Previous 5 contacts	13.7	42.3	33.7	24.0

The subgroups did not differ significantly in these aspects.

### Locum assessment of the importance and quality of the information

Information about the diagnosis, the terminally ill status of the patient, and the patient's medication was regarded as important by almost all locums, as was information about the treatment desired by the patient, relevant changes in the illness process, and the patient's wishes regarding end-of-life care. The prognosis, the patient's awareness of the diagnosis and prognosis, and the psychosocial context were considered to be important information by more than 65% of the locums, and 36.6% considered it important that the GP provided his private telephone number.

In their role as locum, 21.2% of the GPs were satisfied with the quality of the information on terminally ill patients that was available on the GP co-operative, 25.7% were dissatisfied, and 53.1% were neutral with regard to the information available. When asked why they were not satisfied with the information (more than one answer possible), 62.9% stated that it was insufficient, 50% stated that it was not up to date, 48% were dissatisfied because of the absence of information about the terminally ill status of the patients and 20% were dissatisfied because the private telephone number of the patient's GP was not available.

When asked if the transfer of information is a bottleneck the in end-of-life care provided by the GP co-operative, 53.1% considered the transfer of information from GP to GP co-operative to be the most important bottleneck, 37.7% were neutral and 9.1% of all GPs considered it to be unimportant.

The GPs personally available considered information transfer a bottleneck in 56,9% whereas in the group of GPs not often transferring information this was 42%.

### GPs' satisfaction with the feedback report from the locum

In reply to the question about how satisfied they were with the feedback report from the locum at the GP co-operative, 71.5% of the GPs expressed their overall satisfaction. When asked what was missing in the report if they were dissatisfied, 21.7% of the GPs answered that information about "changes in patient treatment" was lacking. Other reasons for dissatisfaction with the locum report were lack of information about: patients and carer's personal situation (17.1%), treatment/medication (9.7%), physical examination (5.7%), medical history (4.6%) and reason for encounter (2.9%).

The GPs personally available reported an overall satisfaction with the feedback report in 65%, whereas in group of GPs often transferring information this was 58.1%.

The GPs were also asked for suggestions to improve the quality of out-of-hours care for terminally ill patients. More than half of the suggestions concerned improvements in the transfer of information.

### Suggestions for improvement of information transfer

• Design a standardised transfer form, to be used as fax form or as e-mail form

• Make direct electronic transfer from GPs electronic file to GP co-operative possible

• Make electronic information available in the locum's car

• Leave a summary of information at the patient's home

• Take down telephone numbers of professionals and carers involved

• Make at least sure that terminally ill patients are known at the GP co-operative

• Update your information regularly

## Discussion and Conclusions

### Main findings of this study

The majority of the GPs in Amsterdam who responded to our questionnaire reported that they transferred information about most of their terminally ill patients to the GP co-operative. However, in their role as locum, the GPs were not satisfied with the quality of the information that was transferred to the GP co-operative. While both the GP and the locum agreed about the importance of transferring explicit clinical data, the locums seemed to value the transfer of information about the patients' personal situation more than GPs. There is consistency between the percentage of GPs who transferred specific clinical data, and the assessment of the importance of this information by the locums. The largest difference between the information transferred and the assessment of its importance is found in the information about knowledge of the patient's personal situation.

The results of this study suggest a difference in views on the transfer of information between the GP in his daily practice and the GP as locum in the GP co-operative. It is possible that GPs over-estimate their performance in transferring information about their terminally ill patients. They do not transfer information as often as they think they do, and the content is not as adequate as they would wish it to be. Since 70% of all GPs stated that they were available for their terminally ill patients during out-of-hours periods, this could be a reason for not transferring information. A reason for under-estimating the importance of transferring information about the patient's personal situation could also be that the GPs did not anticipate a possible deterioration or did not ask about the patient's wishes.

The responses from GPs personally available did differ only slightly from the other respondents: they transferred information almost as much as the other respondents.

The GPs not often transferring information had other characteristics than the other respondents: more male, more single-handed, working four or more days. It looks like they didn't find information transfer important; they work already almost fulltime and didn't transfer information mainly because they were available themselves.

Both groups, GPs personally available and GPs not often transferring information, use the argument of personal availability as reason for not transferring information.

### What is already known on this topic

In the UK, where co-operatives already existed a decade before they were introduced in the Netherlands, the reported problems are similar. GPs are not routinely alerting out-of-hours doctors to the needs of their vulnerable patients [9-11,15]. Important information about two thirds of the patients who were in need of palliative care was not transferred to the co-operative. A major reason was reluctance to define patients as palliative, despite their terminal condition [11].

### Limitations and strengths of this study

The response rate of 42% is relatively low. A possible explanation is that during the data collection a major change in the national health care insurance system took place, which absorbed the GPs' time and energy. Furthermore, the retrospective character of the study may have induced recall bias, and some questions might have evoked socially desirable answers. These factors may have contributed to the large number of GPs who reported that they transferred information and to the GPs' satisfaction with the feedback report from the locum.

A strength of this study is the fact that all GPs were asked to give their view from two different perspectives: as a GP in their daily practice and as a locum in the GP co-operative. The disparity between the two views highlights the inner conflict of the GP who, when busy in daily practice, finds it difficult to write down and transfer the information that he really values when working out-of-hours as a locum. Another strong point is that we approached all GPs in the Amsterdam region, and not only a sample.

## Conclusions

The transfer of information about terminally ill patients to GP co-operatives is often inadequate. Although GPs in Amsterdam reported that they often transferred information, when the same GPs were working as a locum in the GP co-operatives they were unsatisfied with the content of the information that was available for the locum.

GPs consider that continuity of care for their terminally ill patients is a key aspect of the quality of end-of-life care [16]. The rapid development of large-scale GP co-operatives in the Netherlands can be a threat to the transfer of information and continuity, which is highly valued in end-of-life care

## Recommendations

Post-graduate education should focus more on the content of the information that is needed by the locum and train GPs to write adequate (electronic) transfer reports. The use of a standardised transfer form, either as a fax form or e-mail form could be helpful. If an electronic patient file is accessible during the out-of-hours period, this should contain a specific transfer page containing information that is relevant for locums.

Details about the personal situation of these vulnerable patients and of their care needs appear to be of value for the locum.

Moreover, GP co-operatives could develop a systematic procedure for feedback to the GPs about the quality of the information they transfer.

## Competing interests

The authors declare that they have no competing interests.

## Authors' contributions

BS participated in the analysis and drafted the manuscript. NB participated in the draft of the manuscript. MW. EL and PG made up the questionnaire and participated in the analysis. LD helped to draft the manuscript. All authors read and approved the final manuscript.

## Pre-publication history

The pre-publication history for this paper can be accessed here:

http://www.biomedcentral.com/1472-684X/8/19/prepub

## Supplementary Material

Additional file 1 **Questions regarding information transfer**. This file contains all questions used from the questionnaire regarding information transfer. Click here for file
