# Supplementary material for: GPs' views on transfer of information about terminally ill patients to the out-of-hours co-operative
Source: BMC Palliat Care. 2009 Dec 22;8:19. doi: 10.1186/1472-684X-8-19 (PMC2807852; doi:10.1186/1472-684X-8-19)
Supplement: Additional file 1 — Questions regarding information transfer. This file contains all questions used from the questionnaire regarding information transfer. [file 1472-684X-8-19-S1.DOC]

# Additional file 1

# Questions regarding information transfer

**1.** Do you transfer information on a patient to the GPpost when this patient is

terminally ill ?

never always

**2.** If you are not transfering information, what are the reasons for not doing so ?

( more answers possible)

I am always available myself

Patient just dismissed from hospital

Forgotten

Situation deteriorated faster than foreseen

Too much administration

Other reasons :…

**3.** Which information do you transfer to the GPcooperative ?

( more answers possible)

This concerns a terminally ill patient

Diagnosis

Prognosis

List of problems

Medication

Intolerancies

Last five contacts with patient

Relevant changes in the disease process

Patient’s knowledge of diagnosis and prognosis

Wishes regarding patient management

Patient’s wishes regarding end-of-life care

Psychosocial context

Private telephone number of the GP

**4**. Are you on the whole satisfied with the feedback report on your terminally ill

patients from the GPpost ?

never always

**5.** If you are not satisfied with the feedback report, what is missing ?

( more answers possible )

Reason for encounter

Anamnesis

Physical examination

Conclusion of examination

Treatment/Prescribed medication

Changes in patient management

Personal information (wishes patient, carers etc.)

Not applicable

**6.** Are you, as locum, satisfied with the the information on terminally ill patients

transferred by the GP to the GPpost ?

very unsatisfied very satisfied

**7**. If you are, as locum, not satisfied with this information, why not ?

(more answers possible)

There was no information available at all

There was not enough information available

Information was not up to date

No telephone number of patient’s own GP

Not applicable

Other reasons, namely……

**8**. How do you, as locum, assess the importance of the following items when caring

for terminally ill patients at the GPpost ?

1. Diagnosis unimportant very important

2. Terminally ill patient unimportant very important

3. Medication unimportant very important

4. Wishes regarding patient management unimportant very important

5. Prognosis unimportant very important

6. Relevant changes in disease process unimportant very important

7. Patients wishes regarding end-of- life care unimportant very important

8. List of problems unimportant very important

9. Private telephone number GP unimportant very important

10. Patients knowledge of diagnosis and unimportant very important

prognosis

11. Psychosocial context unimportant very important

12. Intolerances for medication unimportant very important

13. Previous 5 contacts unimportant very important

**9**. Do you perceive the following items as bottlenecks when caring for teminally ill patients at the GP cooperative ?

Information transfer never always

Time pressure never always

Difficult communication

( Nursing provision, Farmacy, etc) never always

Difficult to care for unknown

terminally ill patients never always

Patient not aware of possibilities

out-of-hours care never always

Patient not aware of prognosis

and policy never always

Medical policy difficult because of

complexity of problems never always

No anticipatory policy of own GP never always

Own GP not available never always

Other, for example… never always

**10**. Do you have suggestions to improve the quality of out-of-hours care for teminally ill patients ?
